# Impact of 13-valent pneumococcal conjugate vaccine (PCV13) in a pandemic similar to the 2009 H1N1 in the United States

**DOI:** 10.1186/1471-2334-13-229

**Published:** 2013-05-21

**Authors:** Lisa J McGarry, Kristen E Gilmore, Jaime L Rubin, Keith P Klugman, David R Strutton, Milton C Weinstein

**Affiliations:** 1 OptumInsight, One Main Street, Suite 1040, Cambridge, MA, 02142, USA; 2 Department of Global Health, Rollins School of Public Health, Emory University, 1518 Clifton Road, N.E - Room 720, Atlanta, GA, 30322, USA; 3 Pfizer, 500 Arcola Road, Collegeville, PA, 19426, USA; 4 Center for Health Decision Science, Harvard School of Public Health, Harvard University, 718 Huntington Avenue, Boston, MA, 02115, USA

**Keywords:** Pneumococcal disease, Influenza, H1N1 pandemic

## Abstract

**Background:**

High rates of bacterial coinfection in autopsy data from the 2009 H1N1 influenza (“flu”) pandemic suggest synergies between flu and pneumococcal disease (PD) during pandemic conditions, and highlight the importance of interventions like the 13-valent pneumococcal conjugate vaccine (PCV13) that may mitigate the impact of a pandemic.

**Methods:**

We used a decision-analytic model, estimated from published sources, to assess the impact of pediatric vaccination with PCV13 versus the 7-valent vaccine (PCV7) on PD incidence and mortality in a normal flu season (10% flu incidence) and in a pandemic similar to 2009-2010 H1N1 (20% flu incidence, mild virulence, high impact in children). Both direct and indirect (herd) effects against PD were considered. Effectiveness of PCV13 was extrapolated from observed PCV7 data, using assumptions of serotype prevalence and PCV13 protection against the 6 serotypes not in PCV7. To simulate 2009–2010 H1N1, autopsy data were used to estimate the overall proportion of flu deaths with bacterial coinfections. By assuming that increased risk of death during the pandemic occurred among those with comorbidity (using obesity as proxy) and bacterial coinfections primarily due to *S*. *pneumoniae* or *S*. *aureus*, we estimated the proportion co-infected among all (fatal and non-fatal) flu cases (7.6% co-infected with any organism; 2.2% with *S*. *pneumoniae*). PD incidence, mortality, and total healthcare costs were evaluated over a 1-year horizon.

**Results:**

In a normal flu season, compared to PCV7, PCV13 is expected to prevent an additional 13,400 invasive PD (IPD) cases, 399,000 pneumonia cases, and 2,900 deaths, leading to cost savings of $472 M. In a pandemic similar to 2009–2010 H1N1, PCV13 would prevent 22,800 IPD cases, 872,000 pneumonia cases, and 3,700 deaths, resulting in cost savings of $1.0 B compared to PCV7.

**Conclusions:**

In a flu pandemic similar to the 2009–2010 H1N1, protection against the 6 additional serotypes in PCV13 would likely be effective in preventing pandemic-related PD cases, mortality, and associated costs.

## Background

The 1918 influenza pandemic resulted in at least 20 million deaths worldwide, with some estimates as high as 100 million [1-4]. Over the succeeding decades, considerable efforts have been made to determine why mortality during the 1918 pandemic was so high and ensure that such a catastrophic outcome could be avoided in future pandemics. At the time of the pandemic, researchers suspected complications of bacterial pneumonia as the primary driver of excess influenza deaths [5]. This theory has recently gained support from autopsy studies of 1918 influenza victims that have established a link between secondary bacterial infection and death by confirming the presence of bacterial pneumonia in nearly all autopsy samples examined [6,7].

In June 2009, the World Health Organization (WHO) declared the novel influenza A H1N1 virus A/California/7/2009 (referred to as 2009–2010 H1N1) as a pandemic, making it the world’s fourth influenza pandemic in the last century and the first in 40 years [8]. By November 2009, WHO reported more than one-half million confirmed novel H1N1 influenza cases and more than six thousand deaths [9]. Several studies of H1N1-infected patients worldwide have confirmed high levels of bacterial coinfection, and there is evidence to suggest that bacterial coinfection led to excess H1N1 deaths. A retrospective analysis of hospitalized patients with laboratory-confirmed H1N1 in Malaysia found 14 of 50 patients (28%) to have a bacterial coinfection [10], and in a study of 199 H1N1-infected patients in Argentina, *S*. *pneumoniae* was detected in 56% of the 39 H1N1 cases that resulted in hospitalization or death [11]. An autopsy study in Brazil found bacterial coinfection in 8 of 21 (38%) samples [12], an autopsy study of H1N1 victims in New York City confirmed the presence of bacterial pneumonia in 18 of 33 (55%) samples examined for pulmonary bacterial infection [13], and a Centers for Disease Control and Prevention (CDC) H1N1 autopsy study reported evidence of bacterial infection in 22 of 77 (29%) samples [14].

The synergies observed between bacterial pneumonia and influenza with respect to morbidity and death during influenza pandemics suggest pediatric pneumococcal vaccination may mitigate the public health impact of an influenza pandemic by preventing bacterial coinfections with *S*. *pneumoniae*[15]. Since approval of the 7-valent pneumococcal conjugate vaccine (PCV7) in 2000 for pediatric use in children aged up to 59 months [16], PCV7 has resulted in a marked reduction in the incidence of invasive pneumococcal disease (IPD) [17-19] and pneumonia [20] in both the vaccinated and non-vaccinated populations (i.e. due to indirect protection). In a previously published study, we estimated that a fully-implemented PCV7 pediatric vaccination program would avoid 1.2 million cases of IPD and pneumonia and save $7.3 billion in a pandemic of similar severity to 1918 [15].

In 2010, the 13-valent pneumococcal conjugate vaccine (PCV13) replaced PCV7 as the recommended pediatric pneumococcal vaccine [21]. PCV13 is more expensive than PCV7, but protects against the seven serotypes covered by PCV7 plus an additional six serotypes, including 19A. PCV13 vaccination was not in place during the 2009–2010 influenza season, and the possible benefits of the vaccine in a pandemic of this magnitude are unknown. The current study explores the impact of vaccinating children against the additional six serotypes in PCV13 both during a normal influenza season and during a pandemic similar to the 2009–2010 H1N1.

## Methods

### Overview

In a previously-published study, we constructed a decision-analytic model to assess the impact of pediatric PCV7 vaccination versus a policy of no PCV vaccination in the context of an influenza pandemic of 1918 severity [15]. That model was updated to assess the impact of pediatric PCV13 vaccination versus continued vaccination with PCV7 during a normal season [22] and influenza pandemic of 1918 severity (unpublished analysis). For the current analysis, the model was adapted to assess the impact of pediatric PCV13 vaccination versus continued vaccination with PCV7 during an influenza pandemic similar to the 2009–2010 H1N1. Clinical and economic outcomes related to IPD (meningitis and bacteremia) and all-cause pneumonia were evaluated over a 1-year time horizon to reflect one influenza season, and long-term outcomes of events occurring during the one-year period were projected to the lifetime of the model population. Future costs and health outcomes attributable to cases occurring in the index year were discounted to present values using a 3% annual discount rate [23].

The model considers outcomes of pneumococcal disease only; morbidity, mortality, and costs of influenza without pneumococcal coinfection were assumed to be unaffected by pneumococcal vaccination and are not considered. Outcomes include cases of pneumococcal disease, pneumococcal-associated deaths, and related costs. In all analyses, we compared a policy of pediatric PCV13 vaccination to one of pediatric PCV7 vaccination in populations with fully-implemented vaccine programs.

### Estimation

#### Pneumococcal disease incidence and mortality, and vaccine efficacy

Estimates of vaccine coverage, pneumococcal disease incidence and mortality, and vaccine effectiveness have been previously published in a study of the public health and economic impact of PCV13 in the United States (see Appendix) [22]. Briefly, age-specific pneumococcal disease incidence and mortality rates were estimated from Active Bacterial Core Surveillance (ABCs) data [17,24-26] and other published observational studies [20,27-34]. Direct efficacy and expected indirect effects were estimated from published and unpublished age- and serotype-specific data from the ABCs and other published sources [20,24,25,34-38]. Estimates of vaccine efficacy for children aged 2-4 years take into account the waning effectiveness of the vaccine. Indirect effects represent the expected benefit to older children and adults from a fully implemented vaccination program (approximately seven years post-vaccine introduction).

#### Influenza incidence

We estimated the influenza incidence in a pandemic similar to the 2009–2010 H1N1 from data published by the CDC. According to CDC estimates, approximately 61 million US residents were infected with H1N1 during the 2009–2010 influenza season, indicating a cumulative incidence of 20% in a population of approximately 300 million [39]. We estimated age-specific incidence of influenza using data from a study that predicted the true prevalence of H1N1 based on laboratory-confirmed specimens and modeling techniques [40]. We applied the age-specific odds ratios calculated from the prevalence study to the CDC estimates of influenza incidence to arrive at influenza incidence estimates of 44.3% for children aged 0–4 years, 51.7% for the 5–17 years age group, 13.5% for the 18–49 years age group, 7.6% for the 50–64 years age group, and 2.5% for those aged 65+ years. The CDC estimates that approximately 12,470 deaths were associated with the 2009–2010 H1N1, indicating a case-fatality of approximately 0.2% [39].

#### Pneumoco**c**cal disease incidence and mortality in a pandemic

Data on pneumococcal disease incidence during the 2009–2010 H1N1 pandemic are limited. To estimate the incidence of pneumococcal disease and simulate the potential acceleration of pneumococcal disease transmission in a pandemic similar to the 2009–2010 H1N1, we estimated the proportion of influenza cases with bacterial coinfection [14]. Autopsy data were available to estimate the proportion of influenza *deaths* with bacterial coinfection, from which the proportion of coinfection among all H1N1 cases (i.e., both fatal and non-fatal) was derived.

Because the proportion of influenza deaths with coinfection at autopsy is driven both by rates of coinfection and excess mortality due to coinfection, a number of assumptions were necessary to estimate the rate of coinfection among all H1N1 cases. First, we assumed that the underlying increased risk of death due to influenza among those with comorbidity, using obesity as a proxy, was unrelated to coinfection. Second, we assumed that the excess risk of death due to coinfection during the pandemic was equal among those with and without comorbidity. These two assumptions imply that there are no synergies between comorbidity and coinfection with respect to mortality. Third, we assumed that obesity is not associated with risk of influenza or coinfection, such that the prevalence of obesity and morbid obesity among those with influenza is expected to be the same as that in the U.S. population. We further assumed that bacterial coinfections were primarily due to *S*. *pneumoniae* or *S*. *aureus*. Details regarding the equations used to estimate the probability of coinfection are provided in the Appendix. In summary, we derived the following equation for the proportion of H1N1 cases co-infected:

$$\begin{array}{ll} \;\gamma & =\left\{F_{c}\left[1+P_{o}\left(RR_{o}-1\right)+P_{b}\left(RR_{b}-1\right)-1\right]\right\}/\\ & \;\left\{P_{o}\left(RR_{o}-1\right)+P_{b}\left(RR_{b}-1\right)\right\} \end{array}$$

where γ is the probability of bacterial coinfection with any organism among all influenza patients, F_c_ is the fraction of deaths from bacterial coinfection with any organism, P_o_ is the prevalence of obesity, P_b_ is the prevalence of morbid obesity, RR_o_ is the risk ratio for influenza-related death for those obese versus normal weight, and RR_b_ is the risk ratio for influenza-related death for those morbidly obese versus normal weight.

Based on pediatric autopsy data from the CDC, we assumed that 43% of patients dying from 2009–2010 H1N1 influenza were co-infected with any bacterial organism (F_c_), and that 30% of patients with any bacterial coinfection were co-infected with *S*. *pneumoniae*[41]. We estimated the relative risk of death for obese (RR_o_: 3.1) and morbidly obese (RR_b_: 7.6) individuals compared to individuals of normal weight from a case-cohort study of patients hospitalized during the 2009–2010 H1N1 pandemic [42]. Based on obesity data from the National Health and Nutrition Examination Survey (NHANES), we assumed 33.8% of the adult population in the United States to be obese (body mass index [BMI] ≥ 30) and 20% of the obese population to be morbidly obese (BMI ≥ 40) [43]. We then solved for the proportion of all H1N1 influenza patients who also had a bacterial coinfection, resulting in estimates of 7.6% of patients with any bacterial coinfection and 2.2% of patients co-infected with *S*. *pneumoniae*. We used the same model to estimate the risk of bacterial coinfection in a normal influenza season (1.5%). The multiplier on pneumococcal disease incidence during a pandemic influenza season of 5.01 was calculated as the ratio of the pandemic- versus normal-season prevalence of coinfection.

It was assumed that background pneumococcal disease incidence and mortality, as described above in the section on “Pneumococcal Disease Incidence and Mortality, and Vaccine Efficacy”, represent the actual risk of pneumococcal disease during a normal influenza season and therefore any synergies between pneumococcal disease and influenza would be captured in the observed incidence. Therefore, we did not make any additional assumptions about synergies between influenza and pneumococcal disease when estimating the model for a normal influenza season.

#### Costs and utilities

All pneumococcal disease costs were estimated from published sources as described previously; to maintain consistency with previous analyses, costs are in $2008 US [22].Briefly, costs associated with non-hospitalized pneumonia for children aged <5 years and IPD and hospitalized pneumonia for all ages were adapted from Ray et al., 2009 [44]. Costs of long-term consequences of meningitis were adapted from lifetime costs of deafness and disability for children <5 years and adjusted by discounted life-expectancy. Non-hospitalized pneumonia costs for persons aged ≥5 years were estimated as the total of an office visit, chest radiograph, blood culture, and one course of antibiotics [22].

The costs used for PCV7 and PCV13 are consistent with the prices at the time of PCV13 launch as used in previous publications. The average cost for a single dose of PCV13 in the US was assumed to be $100, while the average cost for a single dose of PCV7 was assumed to be $73 [22]. Both vaccines were assumed to have an administration cost of $11 per dose [33].

Utilities, preference-based quality-of-life measures ranging from 0 to 1, were incorporated by assigning a quality-adjusted life-year (QALY) decrement for each pneumococcal disease episode, as described previously [22].Lifetime costs and consequences (including life-years lost and permanent disabilities) attributable to a case of IPD were discounted to present value at an annual rate of 3%.

#### Analyses

The model generates estimates of pneumococcal disease cases avoided, deaths averted, cost differences, and QALYs gained for a policy of PCV13 pediatric vaccination versus a policy of PCV7 pediatric vaccination. Cases and deaths averted were calculated as the difference in the total number of cases/deaths with PCV13 pediatric vaccination minus the total number of cases/deaths with PCV7 vaccination. Cost differences were calculated by subtracting total costs for a policy of PCV13 pediatric vaccination (the cost of vaccinating children with PCV13 less any cost offsets associated with avoiding disease), from total costs associated with a PCV7 pediatric vaccination policy. Analyses were conducted under assumptions meant to replicate the 2009-2010 H1N1 pandemic and also under normal influenza conditions to serve as a comparison to the pandemic. All results are presented from the payer perspective, and therefore consider only direct medical costs.

Direct and indirect effectiveness of PCV13 were estimated by extrapolating from the PCV7 experience, so there is considerable uncertainty regarding the expected benefits of PCV13 in unvaccinated populations. We therefore performed sensitivity analyses to assess how changes in assumptions regarding indirect effectiveness would change model results by, in turn, assuming: 1) PCV13 vaccine has no indirect effectiveness against either IPD or pneumonia, 2) PCV13 vaccine has indirect effectiveness against IPD and hospitalized pneumonia, but no indirect effectiveness against non-hospitalized pneumonia, or 3) PCV13 vaccine has indirect effectiveness against IPD and both hospitalized and non-hospitalized pneumonia. Because our estimation of the prevalence of bacterial co-infection was based on a number of assumptions, we also performed a sensitivity analysis in which the prevalence of co-infection was reduced by half.

## Results

### Base-case

The model predicts that 3.6 million children aged birth to <1 year will receive the pneumococcal vaccine over a 1-year time horizon; an additional 12.2 million children aged 1- < 5 years will receive direct protection from vaccination in prior years. In a normal influenza season, the model predicts that with a PCV7 pediatric vaccination program we would expect 40,800 cases of IPD and 4.52 million cases of all-cause pneumonia; vaccinating children with PCV13 would reduce these numbers to 27,400 IPD cases and 4.12 million all-cause pneumonia cases. In a pandemic similar to the 2009–2010 H1N1, a PCV7 pediatric vaccination policy would lead to an expected 62,100 IPD cases and 9.95 million all-cause pneumonia cases. A policy of PCV13 vaccination reduces this to 39,300 cases of IPD and 9.08 million cases of all-cause pneumonia. Table 1 displays the numbers of additional cases of pneumococcal disease avoided with PCV13 versus PCV7, by age group, for both influenza scenarios.

**Table 1:** Pneumococcal cases avoided with PCV13 vs. PCV7 pediatric vaccination in normal and pandemic influenza seasons

**Normal influenza season**							
	*0*-*1 yrs*	*2-4 yrs*	*5-17 yrs*	*18-49 yrs*	*50-64 yrs*	*65+ yrs*	*Total*
IPD	1,900	1,100	400	3,600	2,500	3,800	13,400
Hospitalized pneumonia	10,000	2,000	3,600	17,500	2,800	22,000	57,400
Non-Hospitalized pneumonia	87,900	34,300	76,400	97,600	12,000	33,000	341,200
Total	99,300	37,400	80,500	118,700	17,300	58,900	412,000
**Pandemic similar to 2009–2010 H1N1**							
	*0-1 yrs*	*2-4 yrs*	*5-17 yrs*	*18-49 yrs*	*50-64 yrs*	*65+ yrs*	*Total*
IPD	5,300	3,100	1,400	5,500	3,300	4,200	22,800
Hospitalized pneumonia	26,400	5,500	11,100	27,000	3,600	24,200	97,700
Non-Hospitalized pneumonia	242,500	94,500	234,600	150,300	15,700	36,300	773,800
Total	274,200	103,100	247,000	182,800	22,600	64,800	894,300

*IPD*, Invasive pneumococcal disease.

**Figure 1:**
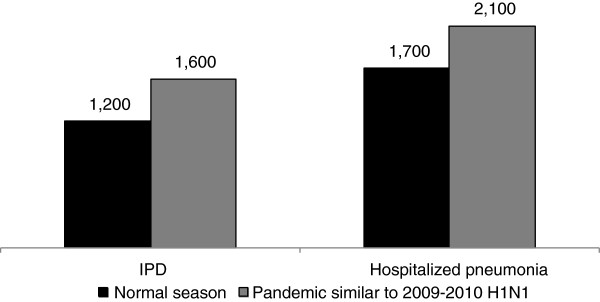
Pneumococcal deaths averted for PCV13 vs. PCV7 pediatric vaccination in normal and pandemic influenza seasons.

In a normal influenza season, PCV13 is expected to prevent an estimated 2,900 deaths compared to PCV7; 4,200 IPD and 23,200 hospitalized pneumonia deaths are expected with PCV7, while 3,000 IPD and 21,500 hospitalized pneumonia deaths are expected with PCV13. For a pandemic similar to 2009–2010 H1N1, PCV13 is expected to prevent an estimated 3,700 deaths compared to PCV7; 5,400 IPD and 27,300 hospitalized pneumonia deaths are expected with PCV7, compared with 3,800 IPD and 25,200 hospitalized pneumonia deaths for PCV13. The numbers of pneumococcal deaths averted for IPD and hospitalized pneumonia for each influenza scenario are presented in Figure 1 (note: discrepancies in the difference calculations are due to rounding).

Vaccination program costs are estimated at $1.1 billion for PCV7 and $1.5 billion for PCV13. In a normal influenza season, pneumococcal disease costs associated with the PCV7 and PCV13 vaccination policies are $7.8 billion and $7.0 billion, respectively. For a pandemic similar to 2009–2010 H1N1, PCV7 and PCV13 vaccination programs are projected to result in pneumococcal disease costs of $12.7 billion and $11.3 billion, respectively. The breakdown of medical cost savings due to vaccination with PCV13, by presentation of pneumococcal disease, is presented in Table 2 for both influenza scenarios.

**Table 2:** Cost savings for PCV13 vs. PCV7 pediatric vaccination in normal and pandemic influenza seasons

**Influenza scenario**	**IPD**	**Hospitalized pneumonia**	**Non-Hospitalized pneumonia**	**Vaccination**	**Total**
Normal season	$ 235 M	$ 509 M	$ 96 M	($ 367 M)	$ 472 M
Pandemic similar to 2009–2010 H1N1	$ 358 M	$ 812 M	$ 213 M	($ 367 M)	$ 1.02 B

*IPD*, Invasive pneumococcal disease; *M*, Million; *B*, Billion.

### Sensitivity analyses

Assuming indirect protection against IPD and no indirect protection against all-cause pneumonia, PCV13 vaccination continues to result in significant cost savings compared to PCV7, for both a normal influenza season and an influenza pandemic season similar to 2009-2010 H1N1. For a pandemic season, if PCV13 is assumed to offer indirect protection only against IPD but not all-cause pneumonia, medical cost savings due to fewer cases of pneumococcal disease nevertheless more than offset the incremental cost of the PCV13 vaccination program compared to PCV7. The cost of the PCV13 vaccination program in a normal influenza season is not entirely offset by medical cost savings under the assumptions of no indirect protection against pneumonia; however, PCV13 vaccination remains cost-effective compared with PCV7 vaccination at a cost of $4,300 per QALY gained. Given the uncertainty in the proportion of all H1N1 influenza patients who also had a bacterial coinfection, the base-case assumption was halved, resulting in a multiplier of 2.5 on pneumococcal disease incidence during a pandemic influenza season. The cost savings associated with the PCV13 vaccination program under this scenario were approximately two-thirds that of the cost savings in the base case. The results of the sensitivity analyses are presented in Table 3.

**Table 3:** Sensitivity analyses around indirect effectiveness of PCV13 and co-infection assumptions in normal and pandemic influenza seasons

**Normal season**				
	*Base-Case*	*IPD indirect effects only*	*No indirect effects*	*Halve % with bacterial co-infection*
**Cases avoided**				
IPD	13,400	13,400	2,100	13,400
Hospitalized pneumonia	57,400	5,000	5,000	57,400
Non-Hospitalized pneumonia	341,200	62,300	62,400	341,200
**Deaths averted**	2,900	1,200	40	2,900
**Cost /(savings)**	$ (472) M	$ 82 M	$ 299 M	($ 472 M)
**QALYs gained**	41,524	18,953	4,484	41,524
**Cost per QALY gained**	dominant	$4,300	$66,800	dominant
**Pandemic similar to 2009–2010 H1N1**				
	*Base-Case*	*IPD indirect effects only*	*No indirect effects*	*Halve % with bacterial co-infection*
**Cases avoided**				
IPD	22,800	22,800	5,700	16,900
Hospitalized pneumonia	97,700	13,700	13,900	72,600
Non-Hospitalized pneumonia	773,800	170,800	171,900	504,000
**Deaths averted**	3,700	1,600	110	3,200
**Cost/(savings)**	($ 1.02 B)	($ 130 M)	$ 180 M	($ 676 M)
**QALYs gained**	51,637	25,671	4,844	44,023
**Cost per QALY gained**	dominant	dominant	$37,100	dominant

*IPD*, Invasive pneumococcal disease; *M*, Million; *B*, Billion; *QALY*, Quality-adjusted life-year.

## Discussion

High rates of bacterial coinfection in autopsy data from the 2009–2010 H1N1 influenza pandemic suggest synergies between influenza and pneumococcal disease in pandemic conditions. While influenza vaccination remains the primary tool to control the significant burden of influenza during both seasonal epidemics and pandemic seasons, pneumococcal vaccination also has the potential to mitigate the impact of pandemic influenza. During the 2009–2010 H1N1 pandemic, protection against the six additional serotypes in PCV13 compared to PCV7 likely would have prevented a portion of the pandemic-related pneumococcal cases, deaths, and costs.

In a typical influenza season, a PCV13 vaccination policy is expected to prevent an additional 13,400 IPD cases, 399,000 all-cause pneumonia cases, and 2,900 deaths, leading to cost savings of $472 million when compared to a PCV7 vaccination policy. We previously estimated that the PCV7 vaccination program in the US was cost saving for a normal influenza season, reducing pneumococcal-related costs by $1.6 billion per year; therefore the total cost savings of PCV13 versus a policy of no pneumococcal vaccination can be estimated at $2.4 billion per year. In a relatively mild pandemic similar to 2009–2010 H1N1, PCV13 is expected to prevent 22,800 IPD cases (1.7x normal season), 872,000 all-cause pneumonia cases (2.2x normal season), 3,700 deaths, and save $1.0 billion (2.2x normal season) compared to PCV7.

Our study is subject to a number of limitations inherent in the study design. First, the decision-analytic model is necessarily a highly simplified representation of the disease transmission and outcomes of pneumococcal disease. Although we accounted for some differences in treatment and outcomes using age stratification, we recognize that the U.S. population and healthcare delivery system are highly heterogeneous and may not be well represented by the relatively simple structure of this model. We also note that data used to estimate vaccine effectiveness and outcomes were derived and synthesized from a variety of sources, and this process of selection and interpretation is subject to bias. Although extensive sensitivity analyses to evaluate the effect of alternative parameter choices on our outcomes showed no change in the overall conclusions, we recognize that different assumptions may have yielded different results.

Costs used in this analysis were taken from published data and standard sources; the extent to which they reflect the true costs of administering medical care is unknown. Furthermore, this study was conducted from a third-party payer perspective rather than a societal perspective, and as such does not include costs of pneumococcal disease related to lost productivity, caregiver time, transportation, or other unreimbursed expenses. Inclusion of these costs presumably would have added substantially to the total cost burden of pneumococcal disease and the potential cost savings with PCV13. In addition, the model was estimated using U.S. data, and care should be used in generalizing our results to other settings and populations.

Because the data available to estimate pneumococcal coinfections were incomplete and based on autopsy data, a number of assumptions were necessary to convert these estimates of pneumococcal infection among influenza deaths to population estimates of coinfection. Although we attempted to be conservative in our assumptions, there remains a high level of uncertainty in these estimates. In particular, our calculations using obesity as a surrogate for all comorbidity is a simplification, and may not reflect the mechanism leading to excess deaths in groups such as those with asthma or immunosuppression, and pregnant women. Studies that examined obesity along with other comorbidities as a risk factor for poor outcomes in H1N1 have shown obesity to be the strongest and most consistent predictor. For example more than half of hospitalized H1N1 cases in California observed between April and August 2009 were obese and one-quarter morbidly obese [45], and obesity was identified as the strongest predictor of death in an analysis of hospitalized H1N1 cases in the United Kingdom (odds-ratio = 6.08; p = 0.01) [46]. We further note, that Morgan and colleagues [42] estimated the odds-ratios of death for obese and morbidly obese persons versus normal weight persons among patients with chronic medical conditions that are risk factors for poor influenza outcomes (as identified by the Advisory Committee on Immunization Practices [ACIP]) and among patients with no chronic medical conditions. They found the odds of death to be significantly elevated only among obese and morbidly obese persons without chronic medical conditions; suggesting that the increased risk associated with comorbidity may be captured by the increase risk due to obesity in patient with both conditions.

It is also possible that our assumption of independence between obesity/comorbidity and bacterial coinfection as causes of death in persons with influenza is not valid. However, we note that there is considerable overlap in the comorbidities considered risk factors for both adverse pneumococcal disease outcomes and adverse influenza outcomes by ACIP [21,47], suggesting that these conditions associated with poor influenza outcome also increase risk in the presence of pneumococcal infection. If there are synergies between comorbidity and coinfection, as suggested by a study in Malaysia [10], the rate of coinfection may be overestimated; however we found that even when we halved our estimate of the rate of coinfection, vaccination remained cost-saving in an assumed pandemic similar to 2009–2010 H1N1.

## Conclusion

Our model suggests that, had PCV13 vaccination been implemented prior to the recent 2009-2010 H1N1 pandemic, the protection provided would have prevented more than 3,500 deaths and saved $1.0 billion. Although the 2009-2010 H1N1 pandemic proved to be far less severe than initial reports suggested, even in a relatively mild pandemic similar to the 2009–2010 H1N1, protection against the six additional serotypes in PCV13 would likely be effective in preventing pandemic-related pneumococcal disease cases, mortality, and associated costs.

## Appendix

This section contains detailed descriptions of model parameters (Table 4) as well as additional explanation around the assumptions and methodology for calculating probabilities of coinfection.

**Table 4:** Parameter Values

**Parameter**	**Age group (years)**					
	**0- < 2**	**2-4**	**5-17**	**18-49**	**50-64**	**65+**
**Annual non-pandemic baseline incidence rates / 100,000**^**a**^						
Pneumococcal meningitis	3.54	0.98	0.27	0.71	1.37	2.39
Pneumococcal bacteremia	33.9	13.2	2.16	6.97	18.5	35.5
All-cause pneumonia	8,749	7,752	1,648	750	328	2,163
**Percent of meningitis that results in deafness**^**b**^	13%	13%	6%	13%	13%	13%
**Percent of meningitis that results in disability**^**b**^	7%	7%	5%	7%	7%	7%
**Case-Fatality Rates**						
Pneumococcal meningitis^c^	6.9%	4.0%	10.0%	10.4%	11.4%	23.8%
Pneumococcal bacteremia^c^	0.9%	0.4%	4.2%	6.2%	11.3%	15.7%
All-cause hospitalized pneumonia^d^	0.4%	0.2%	0.3%	1.2%	2.5%	6.3%
**Direct effects (% reduction in disease at time of vaccination)**^**e**^						
IPD	49.8%	--	--	--	--	--
All-cause hospitalized pneumonia	16.2%	--	--	--	--	--
vAll-cause non-hospitalized pneumonia	3.8%	--	--	--	--	--
**Indirect effects (% reduction in disease)**^**f**^						
IPD	32.7%	39.4%	34.4%	34.5%	24.5%	27.4%
All-cause hospitalized pneumonia	22.5%	0.0%	9.2%	11.3%	8.7%	6.9%
All-cause non-hospitalized pneumonia	6.2%	0.0%	4.7%	5.9%	3.7%	3.4%
**Direct Medical Costs ($2008)**						
Meningitis episode^g^	$17,048	$17,048	--	--	--	--
Bacteremia episode^g^	$3,253	$3,253	--	--	--	--
Cost of invasive disease episode^g^	--	--	$12,738	$17,956	$22,135	$17,216
Lifetime cost of deafness^h^	$96,788	$96,788	$91,663	$73,530	$48,435	$35,261
Lifetime cost of disability^h^	$499,409	$499,409	$472,965	$379,402	$249,915	$181,940
Hospitalized pneumonia episode^h^	$7,276	$7,276	$4,994	$9,248	$10,148	$9,872
Non-hospitalized pneumonia episode^i^	$233	$233	$308	$308	$308	$308
Vaccine Price (per dose)						
PCV7	$73	--	--	--	--	--
PCV13	$100	--	--	--	--	--
Vaccine administration	$11	--	--	--	--	--
**QALY Inputs (all ages)**^**j,k**^	**Meningitis**	**Bacteremia**	**Hospitalized Pneumonia**	**Non-Hospitalized Pneumonia**	**Deafness (utility)**	**Disability (utility)**
	0.023	0.008	0.006	0.004	0.73	0.68

Note: Comprehensive information related to estimation and references is available in two previously published studies [15,22].

a. Incidence rate estimates assume a steady state utilizing 2007 as the baseline year. IPD incidence estimated from unpublished ABCs data provided by Matt Moore [24]. Hospitalized pneumonia incidence estimated from Grijalva [20] with updated estimates for kids <5 years from January 16, 2010 issue of the MMWR. Non-hospitalized pneumonia incidence estimated from analyses of National Ambulatory Medical Care Survey (NAMCS) and National Hospital Ambulatory Medical Care Survey (NHAMCS) 2006 (U.S. Dept. of Health and Human Services, National Center for Health Statistics [27,28].

b. Estimated from Lieu [29] and Shepard [30].

c. Adapted from ABCs Report: Emerging Infections Program Network. *Streptococcus pneumoniae* 2007 and Tsai [32].

d. Fatality rates estimated from National Vital Statistics report [32]; converted to case-fatality rates by applying rate of death to incidence rates from Grijalva et al. [20].

e. IPD estimated from serotype coverage of PCV13 from ABCs data assuming 94% efficacy against those types. Hospitalized pneumonia adapted from PCV7 efficacy in Black [35] adjusted for PCV13 serotype coverage. Non-hospitalized pneumonia adapted from PCV7 efficacy in Hansen [36] adjusted for PCV13 serotype coverage.

f. IPD estimated from serotype-specific prevalence from ABCs data. Pneumonia estimated using age-specific reductions in disease observed after the introduction of PCV7 in 2000, adjusted by serotype coverage.

g. Estimates for all ages adapted from Ray et al. [44].

h. Estimated from Ray et al. [44] for children <5 years; adjusted by relative difference in discounted life expectancy to obtain lifetime costs for older age groups.

i. Estimates for <5 years adapted from Ray et al. [44]; estimates for >5 years assumes 1 physician visit, 46% of patients receive chest radiograph, blood count and culture, and one course of zithromycin.

j. QALY decrements adapted from Melegaro and Edmunds [48], a cost-effectiveness study in England and Wales that synthesized estimates from various primary sources.

k. Utilities for chronic health states estimated from retrospective studies of meningococcal complications [49,50].

### Calculation of Probability of Coinfection

Definition of variables:

γ = probability of coinfection (any organism)

F_c_ = fraction of deaths with coinfection (any organism)*

m_c_ = mortality with coinfection, normal weight

m_o_ = mortality with coinfection, obese

m_b_ = mortality with coinfection, morbidly obese

RR_o_ = relative risk for death, obese vs. normal weight *

RR_b_ = relative risk for death, morbidly obese vs. normal weight *

P_o_ = prevalence of obesity *

P_b_ = prevalence of morbid obesity *

M = total mortality among flu infected

* denotes variables with known or estimated values

Assumptions:

1. All influenza deaths are attributable to either obesity or coinfection.

2. In the absence of coinfection, obese and morbidly obese persons have progressively greater mortality than persons of normal weight.

3. Excess mortality owing to coinfection is the same for obese and non-obese persons.

4. The risks of death associated with coinfection and either obesity or morbid obesity are additive.

5. The incidence of influenza and the prevalence of coinfection among obese and morbidly obese people are the same as for people of normal weight.

### Risk of death

There are 6 groups: 3 obesity categories, each of which can be co-infected or not. The overall probability of death is the weighted average over these 6 groups. Assume those with neither comorbidities nor coinfections are not at risk of death:

(1) $$\begin{array}{ll} \;M= & \gamma \left(1-P_{o}-P_{b}\right)*m_{c}+\gamma P_{o}\left(m_{c}+m_{o}\right)+\gamma P_{b}\left(m_{c}+m_{b}\right)\\ & +\left(1-\gamma \right)P_{o}m_{o}+\left(1-\gamma \right)P_{b}m_{b}\\ & =\gamma m_{c}+P_{o}m_{o}+P_{b}m_{b} \end{array}$$

### Mortality with coinfection

Because we assume that mortality due to obesity is additive to mortality due to coinfection, and that mortality due to coinfection is independent of obesity, the probability of death among persons with coinfection is as follows:

(2) $$A=\left(1-P_{o}-P_{o}\right)*m_{c}+P_{o}\left(m_{c}+m_{o}\right)+P_{b}\left(m_{c}+m_{b}\right)$$

Since the probability of coinfection is further assumed to be independent of obesity status, the probability of dying and being co-infected is γ*Α.

### Fraction of deaths with coinfection

(3) $$\begin{array}{ll} \;F_{c}= & \gamma A/M\\ & =\gamma \left(m_{c}-P_{o}m_{o}+P_{b}m_{b}\right)/\left(\gamma m_{c}-P_{o}m_{o}+P_{b}m_{b}\right) \end{array}$$

### Relative risk (RR) for death, obese vs. normal weight

(4) $$\begin{array}{ll} \;RR_{o}= & \left[\left(1-\gamma \right)m_{o}+\gamma \left(m_{o}+m_{c}\right)\right]/\gamma m_{c}\\ & =\left(m_{o}+\gamma m_{c}\right)/\gamma m_{c} \end{array}$$

### Relative risk for death, morbidly obese vs. normal weight

(5) $$\begin{array}{ll} \;RR_{b}= & \left[\left(1-\gamma \right)m_{b}+\gamma \left(m_{b}+m_{c}\right)\right]/\gamma m_{c}\\ & =\left(m_{b}+\gamma m_{c}\right)/\gamma m_{c} \end{array}$$

### Solution of equations (3)-(5)

$$\begin{array}{ll} \;\gamma = & \left\{F_{c}\left[1+P_{o}\left(RR_{o}-1\right)+P_{b}\left(RR_{b}-1\right)-1\right]-1\right\}/\\ & \left\{P_{o}\left(RR_{o}-1\right)+P_{b}\left(RR_{b}-1\right)\right\} \end{array}$$

(Technical note: We have 3 equations (3)-(5), with 4 unknowns (γ, m_b_, m_o_, and m_c_), but m_b_, m_o_, and m_c_ can only be determined relative to each other – not absolutely - from the data available.)

### Calculation

F_c_ = .43

RR_o_ = 3.1

RR_b_ = 7.6

P_o_ = 0.138

P_b_ = 0.2

$$\begin{array}{ll} \gamma \left(\mathrm{any}\;\mathrm{organism}\right)= & \left\{0.43\left[1+\left(0.138\right)\left(2.1\right)+\left(0.2\right)\left(6.6\right)\right]-1\right\}/\\ & \left\{\left(0.1.38\right)\left(2.1\right)\left(6.6\right)\right\}\\ & =0.1222/1.6\\ & =0.0759 \end{array}$$

Assuming 30% are *S*. *pneumoniae*:

$$\gamma \left(S.\;\mathrm{pneumoniae}\right)=0.0759*.30=0.0228$$

## Abbreviations

ABCs: Active bacterial core surveillance; ACIP: Advisory committee on immunization practices; BMI: Body mass index; CDC: Centers for disease control and prevention; IPD: Invasive pneumococcal disease; NHANES: National health and nutrition examination survey; PCV13: 13-Valent pneumococcal conjugate vaccine; PCV7: 7-Valent pneumococcal conjugate vaccine; WHO: World health organization.

## Competing interests

This study was sponsored by Wyeth, which was acquired by Pfizer Inc. in 2009. DRS is an employee of Pfizer; LJM, KEG, JLR, KPK, and MCW served as paid consultants to Pfizer for this study.

## Authors’ contributions

LJM participated in the study design and methodology, participated in estimating model parameters, and helped to draft the manuscript. KEG performed the model programming and participated in estimating model parameters. JLR participated in the study design and methodology, participated in estimating model parameters and helped to draft the manuscript. KPK provided clinical input to the study design and vetted model parameter estimation. DRS participated in the design of the study and oversaw study methodology and model estimation. MCW was a key contributor to model design and vetted the model structure and analyses. All authors read and approved the final manuscript.

## Pre-publication history

The pre-publication history for this paper can be accessed here:

http://www.biomedcentral.com/1471-2334/13/229/prepub
